# Motivational Themes for Engaging in Randomized Controlled Trials Incorporating Cognitive Training

**DOI:** 10.1093/geroni/igaf122.298

**Published:** 2025-12-31

**Authors:** Jody Nicholson, Maria Vander Meulen, Walter Boot

## Abstract

As of March 2024, the NIH annual budget for Alzheimer’s disease and related dementias (ADRD) research is set to nearly $3.8 billion, after a $100 million increase (Alzheimer’s Association, 2025). This significant increase in funding for cognitive aging research is focused on identifying the causes of age-related cognitive decline and advancing methods and technologies to improve cognitive function in older adults. Whether grant- or industry-funded, clinical trial or observational, two of the most challenging aspects of research are recruitment and retention, which are integral to maintaining validity and generalizability of study results. Computerized cognitive training is one of the most promising non-pharmaceutical interventions for ADRD prevention, so understanding participants’ reasons for engaging in this type of research is crucial to the next stages of implementation research. This symposium highlights research teams who have assessed participant motivation to engage in computerized cognitive training in order understand how motivations for research engagement differ between underrepresented groups (paper 1), how motivation relates to both participant enrollment and drop-out (paper 2), and how to use participant motivation to develop tailored supportive communications in randomized controlled trials to encourage adherence and retention (paper 3). The symposium presents refinement in research examining participant motivation with the studies representing novel contributions given their sample size (paper 1; n = 5,721), link to both a theoretical framework and information on participant withdrawal (paper 2), and a manner in which participant motivation can be strategically applied to increase study retention (paper 3).

